# Emotional Picture Perception: Repetition Effects in Free-Viewing and during an Explicit Categorization Task

**DOI:** 10.3389/fpsyg.2017.01001

**Published:** 2017-07-04

**Authors:** Serena Mastria, Vera Ferrari, Maurizio Codispoti

**Affiliations:** ^1^Department of Psychology, University of BolognaBologna, Italy; ^2^Department of Medicine and Surgery, University of ParmaParma, Italy

**Keywords:** emotion, repetition, categorization, late positive potential

## Abstract

Several studies have found that, despite a decrease in the overall amplitude of the late positive potential (LPP) with repeated presentation of the same picture, emotional stimuli continue to elicit a larger LPP than neutral ones. These findings seem to support the hypothesis that the affective modulation of the LPP reflects a mandatory process and does not rely on stimulus novelty. However, in these studies participants were asked to merely look at the pictures, without carrying out any additional task (free-viewing), making picture emotionality the most salient aspect of the stimulus, despite its repetition. The current study aimed to examine the impact of an explicit categorization task on the emotional processing of repeated pictures. To this purpose, ERPs to novel and repeated pictures were measured during free-viewing as well as during an explicit categorization task, where the emotional content of the pictures was task-irrelevant. The within-subject comparison between the free-viewing and task context revealed that the overall LPP habituated more rapidly in the free-viewing condition, but, more importantly, the LPP affective modulation was unaffected by task requirements during both novel and repeated presentations. These results suggest that the affective modulation of the LPP reflects an automatic engagement of cortico-limbic motivational systems, which continues to take place regardless of stimulus novelty and task context.

## Introduction

Our perceptual system serves the adaptive function of identifying features in the sensory environment that might be of potential relevance to behavior. While most scenes, people, or objects encountered everyday can be ignored, some of them elicit patterns of brain responses because of their intrinsic *stimulus significance*. Such stimuli include emotional stimuli (e.g., threatening or appetitive cues) and novel stimuli.

Emotional processing has been widely investigated by presenting affective pictures that are effective cues in evoking a broad range of emotional reactions, varying in intensity, and involving both pleasant and unpleasant affect (Lang and Bradley, 2010). One of the most well-established findings in the literature on affective picture processing is that emotionally arousing (pleasant and unpleasant) pictures elicit a larger late positive potential (LPP) than neutral pictures (Radilová, 1982; Johnston et al., 1986; Dolcos and Cabeza, 2002; Codispoti et al., 2006b; Schupp et al., 2006; Bradley, 2009; Hajcak et al., 2012), which seems to reflect both the engagement of attentional resources by emotional stimuli and the activation of cortico-limbic appetitive and defensive systems.

Previous studies found that during explicit categorization tasks (Ferrari et al., 2008), in which individuals directed attention to target stimuli that had been defined according to a specific semantic category (e.g., animals), the emotional modulation of the LPP was always evident even when affective stimuli were presented as distractors (i.e., non-target pictures), suggesting that the evaluative process underlying the LPP affective modulation does not depend on task instructions.

Similarly, when attention was manipulated by stimulus repetition, both pleasant and unpleasant pictures continued to elicit larger LPPs than neutral pictures even after massive repetition of the same stimulus exemplars (Codispoti et al., 2006a, 2007; Ferrari et al., 2013, 2015, 2016). On the other hand, the overall amplitude of the LPP was attenuated across repetitions, and this is consistent with the hypothesis that fewer resources were allocated to pictures as their novelty and salience declined. However, considering that most of the repetition studies were performed in a free-viewing context, in which participants were simply asked to look at the pictures without an explicit concurrent task (Codispoti et al., 2006a, 2007; Ferrari et al., 2011), we cannot rule out the hypothesis that, despite repetitions, attention was voluntarily directed toward categorizing emotional picture content among several neutral stimuli, thus helping to maintain the LPP affective modulation. Moreover, previous studies did not compare a free-viewing context with an explicit task.

In the present study, we aimed to examine the impact of an explicit categorization task on the emotional processing of repeated pictures. To this purpose, picture repetitions (both emotional and neutral) occurred either in a free-viewing context or during a categorization task (within-subject design), with pictures depicting any means of transportation serving as targets, and pictures depicting contents other than means of transportation (including pleasant and unpleasant cues) serving as non-target stimuli.

When repetition occurred in a free-viewing context, emotional cues were the most salient aspect of the stimulus to process. Thus, in the present study we may expect a more evident impact of repetition on the affective modulation of ERPs when participants were engaged in a categorization task in which attention was not specifically directed toward affective information, compared to when repetitions occurred in a free-viewing condition. On the other hand, an opposite scenario could be predicted based on early oddball studies (Polich and McIsaac, 1994; Bennington and Polich, 1999), which showed that habituation of the late positivity prompted by rare stimuli was more evident in passive (i.e., free-viewing) paradigms, compared with active discrimination tasks. Thus, following these findings, we could also expect that the introduction of a task context, which explicitly requires picture processing, prompts a slower habituation compared to a free-viewing context. The absence of a task effect on the LPP affective habituation, on the other hand, would be consistent with the interpretation that the LPP elicited by emotional stimuli reliably reflects a mandatory evaluation of their motivational significance. All these scenarios will provide constructive information regarding emotional processing and affective habituation.

## Materials and Methods

### Participants

Participants were 33 students (19 females) from the University of Bologna who volunteered to take part in the study. All participants had normal or corrected to normal vision, and none of them reported current or past neurological or psychopathological problems. They had no previous experience with the materials used in this experiment. The experimental protocol conformed to the Declaration of Helsinki and was approved by the Bioethics committee of the University of Bologna. Due to technical problems, one female participant was excluded from behavioral analyses, and three participants (two males and one female) were excluded from the EEG analysis.

### Stimuli

The visual stimuli were 252 color pictures of natural scenes selected from various sources, including the International Affective Picture System (IAPS; Lang et al., 2008), and public-domain pictures available on the Internet, depicting people in pleasant (erotic and romantic couples), neutral (portrait or multiple people in a daily context), and unpleasant (mutilated bodies and human attack) contexts. These emotional categories were selected based on previous studies showing that they are the most effective contents in eliciting physiological changes, and do not differ in terms of arousal as defined using: (a) subjective ratings and (b) autonomic changes (Bradley et al., 2001, 2003; Schupp et al., 2004a; Codispoti and De Cesarei, 2007; De Cesarei and Codispoti, 2008). The number of picture exemplars was equally distributed across picture contents. The average valence and arousal scores of the selected IAPS pictures^1^ were, respectively (numbers in parentheses are standard deviations), 6.7 (0.49) and 6.0 (0.71) for pleasant pictures, 5.2 (0.63) and 3.5 (0.41) for neutral pictures, and 2.2 (0.77) and 6.1 (0.80) for unpleasant pictures (Lang et al., 2008). A one-way analysis of variance (ANOVA) on the valence scores of pleasant, neutral, and unpleasant pictures yielded a significant main effect of valence, *F*(2,164) = 742.1, *p*s < 0.0001, $\eta_{p}^{2}$ = 0.90, with significant differences between all three stimulus categories (all comparisons *p*s < 0.0001). A second ANOVA on the arousal scores yielded a significant main effect of arousal, *F*(2,164) = 169.2, *p*s < 0.0001, $\eta_{p}^{2}$ = 0.67. Emotional pictures were rated as more arousing than neutral pictures (both *p*s < 0.0001), with no difference between pleasant and unpleasant pictures (*p* = 0.21). In addition, 280 pictures depicting a variety of means of transportation (e.g., trains, cars, and airplanes) were selected from public-domain images available on the Internet. All pictures were equated in brightness and contrast (155 and 25 on a scale ranging from 0 to 255), using the gamma parameter of the Weibull fit (Yanulevskaya and Geusebroek, 2009). On the other hand, it is worth mentioning here that we focused our ERP analysis on the LPP component, which has been suggested to reflect emotional processing of the semantic, rather than perceptual, properties of the stimuli (Bradley et al., 2007; De Cesarei and Codispoti, 2011b; Codispoti et al., 2012).

### Design and Procedure

**FIGURE 1:**
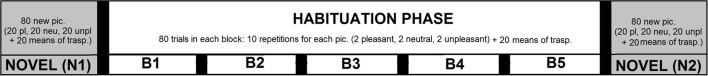
Schematic experimental structure depicting the sequence of blocks in the present study. The experimental session started with a block of all novel pictures (N1), depicting people in pleasant, neutral, and unpleasant context, as well as pictures depicting means of transportation. Across the five blocks of the habituation phase (B1 to B5), 2 pleasant, 2 neutral, and 2 unpleasant pictures were repeated 50 times. After the habituation phase, a final block of only novel pictures (N2) was presented. Pictures of means of transportation were never repeated throughout the experiment.

For each participant, two different sets of pictures were selected to be presented in two different experimental sessions, that took place one after the other, within the same day. The experimental structure (see **Figure 1**) was identical between the two sessions. In each session, pictures were arranged in seven blocks of 80 trials each. In each block, 20 pictures belonged to the category of means of transportation, whereas the remaining 60 trials were equally representative of pictures depicting people in pleasant, neutral, and unpleasant contexts. A series of five blocks were presented that constituted the habituation phase (B1 to B5), during which 2 pleasant, 2 unpleasant and 2 neutral pictures were repeated 10 times in each block (a total of 50 repetitions). Two blocks of only novel pictures were presented (i.e., novel blocks, N1 and N2), respectively, before and after the habituation phase. Pictures of means of transportation were always novel pictures throughout the study, as the same exemplar was never repeated.

In one session participants were required to perform a categorization task, indicating whether the presented image contained a means of transportation (target) or not. In this session (i.e., task context), pictures of people served as non-target stimuli. In the other session, participants were only required to simply look at each picture without an explicit response task (i.e., free-viewing context), such that there was not an instructed discrimination among picture categories.

The specific set of pictures serving as novel or repeated varied across participants, such that, across subjects, all pictures were used in all conditions. The order of picture presentation was pseudo-randomized with the restriction that no more than two times consecutively the same picture or a picture of the same valence occurred.

After completing the informed consent form, participants were seated in a chair in a sound-attenuated, dimly lit room, and the EEG sensor net was attached. In the categorization task, participants were instructed to respond whether the presented image contained the target category (any means of transportation) or not, by pressing one of two buttons on the keyboard (“z” or “m”) as fast and accurately as possible. For half of the participants, the letter-button mapping was reversed. In the free-viewing context, participants were instructed that a series of pictures would be presented and that each picture should be viewed the entire time it was on the screen. Half of the participants performed the categorization task at first followed by the free-viewing condition; the order was reversed for the other half. Between the two sessions there was a 15 min-break. Participants sat in front of a computer monitor with their head supported by a chinrest. The distance between the eyes and the monitor was 100 cm for all subjects. Stimuli were presented on a 21″ CRT monitor at 800 × 600 pixel-resolution and 85 Hz refresh rate, subtending 22.6° horizontal × 17.1° vertical degrees of visual angle. E-Prime software (Schneider et al., 2002) synchronized the presentation of the stimuli and triggered EEG recording on each trial. During each trial, each picture was presented and remained visible for 1 s. After picture offset, a gray screen was displayed for an amount of time ranging from 1500 to 2500 msec (intertrial interval, ITI).

### EEG Recording and Data Reduction

EEG was recorded at a sampling rate of 512 Hz from 256 active sites using an ActiveTwo Biosemi system. An additional sensor was placed below participant’s left eye, to allow for detection of blinks and eye movements. The EEG was referenced to an additional active electrode located near Cz during recording. Off-line analysis was performed using Emegs (Peyk et al., 2011). EEG data were initially filtered (40 Hz low-pass), and eye movements were corrected by mean of an automated regressive method (Schlögl et al., 2007). Trials and sensors containing artefactual data were detected through a statistical procedure specifically developed for dense array EEG (statistical correction of artifacts in dense array studies, SCADS; Junghöfer et al., 2000). Trials containing a high number of neighboring bad sensors were discarded; for the remaining trials, sensors containing artefactual data were replaced by interpolating the nearest good sensors. Then, data were re-referenced to the average of all sensors, and a baseline correction based on the 200 msec prior to stimulus onset was performed. Based on previous studies (e.g., Cuthbert et al., 2000; Codispoti et al., 2006b), and on the maximal amplitude of the differential activity between emotional (average of pleasant and unpleasant) and neutral pictures during the free-viewing context, the LPP amplitude was calculated in the time interval 300–600 msec at centro-parietal sensor sites.

### Data Analysis

The LPP amplitude during picture viewing was submitted to a repeated-measures analysis of variance (ANOVA) with Picture Content (four levels: pleasant, neutral, unpleasant, and means of transportation), Block (seven levels: one block of novel pictures, five consecutive blocks of repeated pictures, and a final block of only novel pictures), Task (two levels: free-viewing, categorization task), as within-subject factors. Greenhouse-Geisser corrections were applied where relevant. The partial eta squared statistic (η^2^ p), indicating the proportion between the variance explained by one experimental factor and the total variance, has been calculated and is reported. During the categorization task, analysis of reaction times (RTs) was performed only on trials associated with correct responses. Responses were scored as correct if the appropriate response latencies were not shorter or longer than two standard deviations from the individual mean. Mean RTs and Accuracy scores (%) were analyzed in a two-way ANOVA with Block and Picture Content as within-subject factors.

## Results

### Late Positive Potential

**FIGURE 2:**
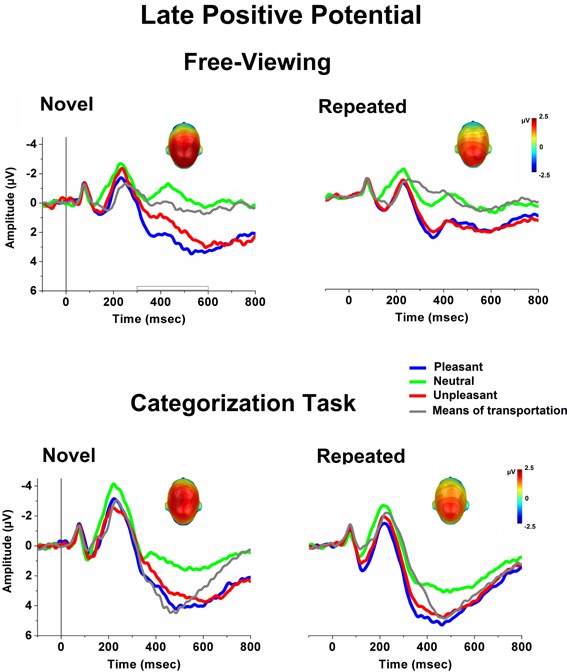
Grand-Average ERPs (over centro-parietal sensors) elicited when viewing novel and repeated (average of 50 repetitions across the habituation phase) pictures in free-viewing (upper panel) and during a categorization task (lower panel), for each picture category. In the categorization task, pictures depicting means of transportation served as target stimuli. Insets are the top view of the scalp distribution of the difference in the 300–600-ms window between emotional and neutral picture processing [i.e., the late positive potential (LPP) affective modulation].

**Figure 2** illustrates ERPs (averaged over centro-parietal sensors) elicited when viewing novel and repeated (average of 50 repetitions of the same picture exemplar) pictures in free-viewing (upper panel) and during a categorization task (lower panel). A striking difference between the free-viewing and the task condition was in terms of the overall ERP amplitude (i.e., larger N2 and P3) which was much more emphasized during the task context, perhaps reflecting the recruitment of different brain regions, or an amplitude change in a subset of those generators between the two experimental conditions (Johnson, 1988). In addition to this general and expected effect of the task on the overall ERP waveform, stimulus-specific modulatory effects were observed as a function of task relevance, and were particularly evident in the LPP component over the centro-parietal scalp region. As illustrated in **Figure 2**, during the free-viewing condition, unpleasant and pleasant stimuli elicited larger LPPs compared to neutral people, as well as to pictures of means of transportation. During the categorization task, pictures of means of transportation, which served as target stimuli, were associated with LPP amplitudes which were more similar to what observed for emotional stimuli. These cortical modulatory effects were nearly preserved, despite an overall reduction in the LPP amplitude across stimulus repetitions.

Main effects of Content, *F*(3,87) = 66, *p* < 0.0001, $\eta_{p}^{2}$ = 0.7, Block, *F*(6,174) = 10, *p* < 0.0001, $\eta_{p}^{2}$ = 0.26, and Task, *F*(1,29) = 40, *p* < 0.0001, $\eta_{p}^{2}$ = 0.58 were further qualified by the following interactions, Content × Task, *F*(3,87) = 13.6, $\eta_{p}^{2}$ = 0.32, Content × Block, *F*(18,522) = 5, *p* < 0.0001, $\eta_{p}^{2}$ = 0.15, and Block × Task, *F*(6,174) = 8.6, *p* < 0.0001, $\eta_{p}^{2}$ = 0.23.

The interaction Content × Task was mainly due to the effect of task relevance on the LPP prompted by pictures of means of transportation. Compared to the free-viewing condition, the task prompted a centro-parietal positivity enhancement, which was larger for pictures depicting means of transportation, *F*(1,29) = 48, *p* < 0.0001, $\eta_{p}^{2}$ = 0.62, that served as target stimuli, compared to non-target pictures (pleasant, unpleasant, and neutral pictures, *F*s(1,29) > 17, *p*s < 0.0001, $\eta_{p}^{2}$ = 0.37). More specifically, the LPP amplitudes elicited when viewing pictures of means of transportation in free-viewing was significantly smaller compared to the LPP to pleasant and unpleasant pictures, *F*(1,29) > 63, *p*s < 0.0001, $\eta_{p}^{2}$ > 0.68, and did not differ from the LPP to pictures of neutral people (*F* < 1). When instead means of transportation served as targets, the LPP amplitude was significantly larger compared to non-target neutral people, *F*(1,29) = 29, *p* < 0.0001, $\eta_{p}^{2}$ = 0.5, and did not differ from the LPP prompted by non-target unpleasant pictures (*p* > 0.05). The affective modulation of the LPP, with larger positive amplitude during viewing of both pleasant and unpleasant, compared to neutral, pictures, was not affected by the viewing context (free-viewing: pleasant, *F*(1,29) > 155, *p* < 0.0001, $\eta_{p}^{2}$ = 0.8; unpleasant, *F*(1,29) = 77, *p* < 0.0001, $\eta_{p}^{2}$ = 0.7; categorization task: pleasant, *F*s(1,29) = 135, *p* < 0.0001, $\eta_{p}^{2}$ = 0.8; unpleasant, *F*s(1,29) = 69, *p* < 0.0001, $\eta_{p}^{2}$ = 0.7). Pleasant pictures prompted an overall larger positivity compared to unpleasant pictures, *F*(1,29) = 9, *p* < 0.01, $\eta_{p}^{2}$ = 0.24.

**FIGURE 3:**
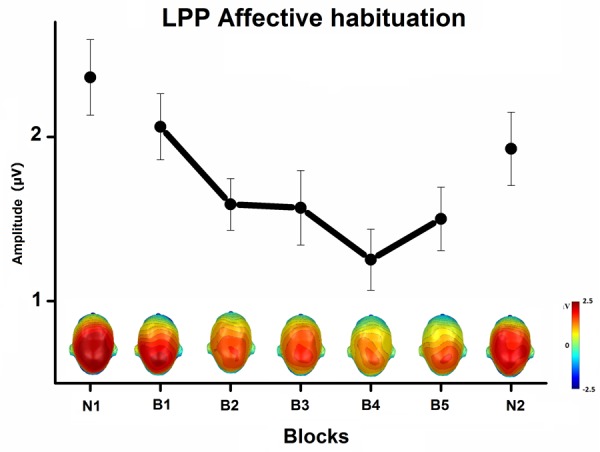
Mean LPP affective modulation (pictures of emotional minus neutral people) over centro-parietal sensors, and the top view of scalp topography, in the 300–600-ms window, across blocks of novel and repeated pictures (N1, first novel block; B1 to B5, habituation phase; N2, last novel block), averaged across task and free-viewing contexts. Error bars represent one standard error of the mean.

The Content × Block interaction showed the habituation of the LPP affective modulation over blocks of repeated pictures (**Figure 3**). The difference in the LPP amplitude between emotional and neutral pictures significantly decreased with picture repetition [B1 to B5, *F*(8,232) = 3, *p* < 0.05, $\eta_{p}^{2}$ = 0.09], but remained highly significant until the end of the habituation phase, that is after 50 repetitions of the same exemplar [B5, quadratic trend, *F*(1,29) = 54, *p* < 0.0001, $\eta_{p}^{2}$ = 0.65]. More specifically, the LPP amplitude decreased with picture repetition only during the viewing of emotional pictures [B1 to B5, *p* < 0.001], but remained stable during the viewing of neutral pictures. In the novel phase that followed blocks of repeated pictures, the LPP was equivalent in magnitude to that elicited in the first novel phase, with emotional pictures again prompting a larger LPP than neutral pictures, quadratic trend *F*(1,29) = 74, *p* < 0.0001, $\eta_{p}^{2}$ = 0.72.

**FIGURE 4:**
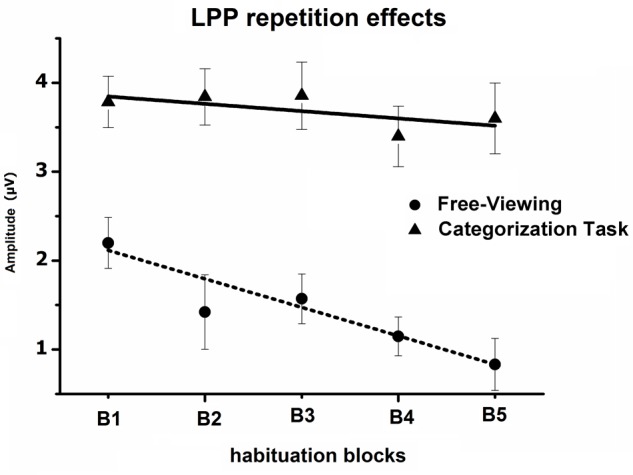
Mean overall LPP amplitude over centro-parietal sensors in the 300–600-ms window, regardless of picture content, across the five blocks of the habituation phase, during both the free-viewing and categorization task. Error bars represent one standard error of the mean.

The Block × Task interaction indicated that the overall impact of picture repetition was modulated by the task (see **Figure 4**), as the linear decrease in the LPP amplitude across blocks of repeated pictures (B1 to B5) was less pronounced when participants were engaged in the categorization task, compared to the free-viewing condition [linear by linear, *F*(1,29) = 7.7, *p* < 0.05, $\eta_{p}^{2}$ = 0.21]. On the other hand, the habituation of the LPP affective modulation did not differ between the free-viewing and the categorization task (three-way interaction, *p* > 0.05) (**Table 1**).

**Table 1:** Means (standard errors) of the late positive potential (microvolts) for each Picture Content, Block, and Context.

		Pleasant		Neutral		Unpleasant		Means of transp.	
					
		Free	Task	Free	Task	Free	Task	Free	Task
Novel phase	N1	2.4 (0.3)	2.7 (0.4)	-0.5 (0.3)	0.4 (0.3)	1.9 (0.4)	2.2 (0.4)	-0.1 (-0.5)	2.2 (0.4)
	N2	2.6 (0.3)	3.5 (0.5)	-0.4 (0.3)	1.5 (0.3)	1.2 (0.3)	2.5 (0.4)	0.5 (0.3)	3.8 (0.4)
Habituation	B1	2.9 (0.4)	4.8 (0.4)	0.7 (0.3)	2.4 (0.4)	2.9 (0.4)	4.2 (0.3)	0.3 (0.4)	2.9 (0.4)
	B2	1.8 (0.5)	4.4 (0.4)	0.4 (0.3)	2.7 (0.3)	2.1 (0.5)	4.5 (0.4)	0.4 (0.3)	3.6 (0.4)
	B3	1.9 (0.4)	4.5 (0.4)	0.3 (0.4)	3.0 (0.4)	2.5 (0.3)	4.1 (0.4)	0.2 (0.5)	3.4 (0.4)
	B4	1.9 (0.2)	4.2 (0.4)	0.4 (0.3)	2.5 (0.3)	1.2 (0.4)	3.5 (0.4)	0.0 (0.4)	3.6 (0.4)
	B5	1.4 (0.3)	4.6 (0.5)	-0.0 (0.3)	2.3 (0.4)	1.1 (0.5)	3.9 (0.4)	0.0 (0.3)	3.7 (0.5)


### Behavioral Interference in the Categorization Task

**FIGURE 5:**
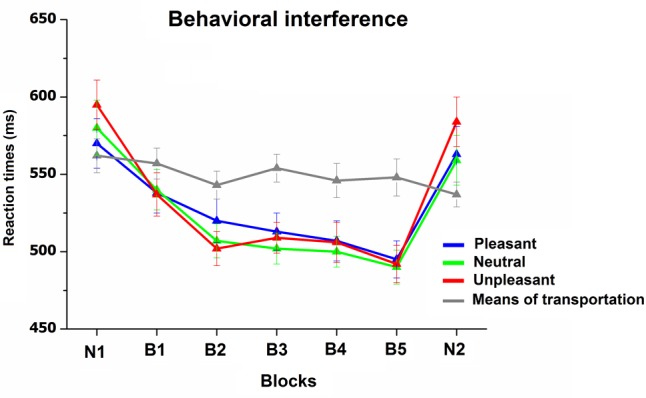
Reaction times (RT) to targets (means of transportation) and non-targets (pleasant, neutral, and unpleasant) during the categorization task. Error bars represent one standard error of the mean.

**Figure 5** illustrates RTs to target and non-target pictures during the categorization task. RT analysis revealed a main effect of Block, *F*(6,180) = 29, *p* < 0.0001, $\eta_{p}^{2}$ = 0.49, Content, *F*(3,90) = 7.1, *p* < 0.01, $\eta_{p}^{2}$ = 0.19, as well as a significant interaction Block × Content, *F*(18,540) = 18, *p* < 0.0001, $\eta_{p}^{2}$ = 0.38. The interaction indicated that, whereas RTs responses to target stimuli, which were always novel scenes throughout the study, remained stable across blocks, RTs to non-target stimuli (pleasant, neutral, and unpleasant scenes) decreased as pictures were repeated throughout the habituation phase. Following the habituation phase, the viewing of novel pictures prompted a significant RT slowdown for all non-target pictures [B5 vs. N2, *F*s(1,31) > 43, *p* < 0.0001, $\eta_{p}^{2}$ > 0.58], which was similar to what observed in the initial novel block [Block_(2)_ × Content_(3)_, *F* < 1]. The emotional content of the pictures was effective in modulating RTs only during the viewing of novel pictures (both N1 and N2), with slower RTs for unpleasant, compared to neutral and pleasant pictures, *F*s(1,31) > 27, *p* < 0.0001, $\eta_{p}^{2}$ > 0.4, with no difference between neutral and pleasant pictures. More relevant, this emotional RT modulation disappeared as soon as pictures were repeated (B1, *F* < 1). The overall accuracy during the categorization task was high (average 98%), and no main effect or interaction was significant.

## Discussion

The free-viewing of emotional pictures is typically associated with a larger positive potential (LPP) measured over centro-parietal sites, compared to when pictures depict neutral contents, and this cortical modulatory pattern is only moderately affected by stimulus repetition, even with a high number of repetitions of the same stimulus exemplars (Codispoti et al., 2007). In the current study, the introduction of a categorization task, during which repeated emotional pictures were non-targets, was aimed at investigating the role of top-down processes in the persistence of the LPP affective modulation over repetitions.

A within-subject comparison between free-viewing and categorization task conditions revealed stimulus-specific modulatory effects as a function of task relevance, that were particularly evident in the LPP component over the centro-parietal scalp region. Pictures depicting means of transportation elicited larger LPPs when they were target stimuli, compared to when they were simple neutral stimuli in the free-viewing condition, where they prompted similar LPP amplitudes to those measured for other neutral contents (e.g., people). Moreover, consistent with previous studies (Ferrari et al., 2008), the LPP amplitudes were similar between target stimuli and non-target emotional pictures, indicating that irrespectively of whether a visual stimulus is task-relevant (target) or is simply affectively engaging, similar cortical differences, as measured by ERPs, are found over centro-parietal sensors.

The impact of the task was also evident on the rate of habituation of the overall centro-parietal positivity, which was somewhat milder compared to that of the free-viewing condition. This overall pattern of results is consistent with several oddball studies that investigated the P3 component during active and passive (or ignore) conditions (Courchesne, 1978; Polich, 1987; Kenemans et al., 1988; Romero and Polich, 1996; Friedman et al., 1998; Bennington and Polich, 1999). According to the context or memory-updating model of P3 amplitude, the size of this ERP component is directly proportional to the amount of change required to update the active memory representation of the stimulus environment when a new stimulus is processed (Donchin, 1981; Verbaten et al., 1986; Kenemans et al., 1988; Polich, 2007). Consistent with these findings, in the present study the rate of habituation of the LPP was less pronounced during an active task, compared to a free-viewing condition, probably reflecting changes in the amount of attentional resources being allocated to process the target stimulus when memory is updated.

On the other hand, while the task affected the overall centro-parietal LPP and its reduction across repetitions, the within-subject comparison between the free-viewing and task context revealed that the LPP affective modulation, and its slight habituation, were similar across the two conditions. The novel phases of the present study, in which only new pictures were presented, also allowed us to evaluate whether a free-viewing context weakened the impact of emotional pictures, as measured by ERPs, compared to when participants directed attention to picture content in order to perform a decision task. Some previous studies have compared the impact of different types of task contexts on the LPP affective modulation (Ferrari et al., 2008; Weinberg et al., 2012; Gable et al., 2015), mostly showing additive effects of task instruction and affective content on the LPP modulation. Here we show that the LPP affective modulation was as large and reliable in the free-viewing condition as it was during a categorization task, proving once again how pictures of natural scenes are effective cues in activating motivational (appetitive and aversive) circuits. However, different findings were reported when the task required to focus spatial attention on a different visual stimulus. Schupp et al. (2014) asked participants to perform a visual categorization task (Thorpe et al., 1996), where small task images were briefly (26 ms) presented and centrally overlaid upon larger emotional or neutral background pictures (Schupp et al., 2014). Additionally, participants passively viewed the same stimulus materials without the demand to categorize task images. While in the passive viewing condition emotional background elicited an affective modulation of the LPP, during the categorization task, the emotional content of the background pictures did not affect the LPP amplitude. The authors interpreted these findings suggesting that the categorization task interfered with emotional processing when both processes compete for shared resources (Vuilleumier, 2005; Pessoa, 2008; Shafer et al., 2012). Moreover, it is worth noting that the absence of LPP affective modulation for the background scenes might also be due to narrowing of the spatial attention window around the smaller target pictures, effectively excluding stimuli outside it. Similarly, Shafer et al. (2012) found evidence that although novel emotional stimuli engage attentional resources even when they are distractors, and briefly presented, emotion effects were influenced by the amount of available attentional resources (Erthal et al., 2005; Okon-Singer et al., 2007; Shafer et al., 2012; Iordan et al., 2013).

Previous studies on stimulus repetition found that while participants were asked to simply look at the pictures (free-viewing context) the affective modulation of the LPP was only slightly affected by picture repetition, and persisted even after massive repetition (Ferrari et al., 2011, 2017b). In the present study, the affective habituation of the LPP was similar between the free-viewing and task context, showing that the presence of a categorization task, which implied enhanced attention allocation toward semantic picture processing, did not prompt any effect on the LPP affective modulation across repeated pictures. In other words, manipulating the amount of attention toward the visual stimuli neither increased nor decreased the impact of picture repetition on the LPP affective modulation, indicating that the persistence of the LPP affective modulation over repetitions is a mandatory process, in the sense that it does not rely on attentional allocation involved in picture processing.

Behavioral data also provided the opportunity to investigate repetition effects on behavioral interference triggered by emotional stimuli. As expected, when pictures were novel, RTs were slower during the viewing of unpleasant, compared to neutral, pictures, showing an attentional capture effect (Hartikainen et al., 2000; De Cesarei and Codispoti, 2008; Weinberg and Hajcak, 2011; Ferrari et al., 2014, 2017a; Padmala and Pessoa, 2014; Calvo et al., 2015). More relevantly, the present study showed that, unlike the LPP, the interference effect rapidly declined with stimulus repetition. Importantly, novel pictures presented after the habituation phase prompted a full recovery of RT modulation, indicating that the decline of emotional interference across repetitions did not reflect a general habituation to task-irrelevant emotional stimuli but, rather, was stimulus-specific. The present results therefore demonstrate that emotional stimuli continue to engage motivational systems even when behavioral emotional interference on the primary task is inhibited. These findings are consistent with a previous study showing that affective modulation of the LPP was preserved despite picture repetition, even when emotional pictures were task-irrelevant distractors, and no behavioral emotional interference was observed (Codispoti et al., 2016). Results from both studies support the hypothesis that evaluative processing, and the engagement of the motivational systems, may occur independently of attentive processes.

In the present study, pleasant and unpleasant pictures elicited a larger LPP compared to neutral stimuli consistent with several previous studies (Dolcos and Cabeza, 2002; Codispoti et al., 2006a, 2007; Schupp et al., 2006; Bradley, 2009; Lang and Bradley, 2010; Hajcak et al., 2012). In addition, we found a slightly larger LPP for pleasant pictures compared to unpleasant stimuli. Although this effect has been reported in some previous studies using similar picture contents (erotica/romance and violence/mutilation), which were balanced in terms of arousal ratings (Schupp et al., 2004a; Briggs and Martin, 2009; Wiens and Syrjänen, 2013), other studies found no LPP differences (Schupp et al., 2004b, 2007; Codispoti et al., 2006a, 2007; De Cesarei and Codispoti, 2011a). One explanation for these mixed results might be related to differences across studies in the composition of the sample in terms of sexual attitudes and sexual desire (Prause et al., 2015).

In summary, these findings indicate that evaluative processes strongly engage the motivational systems during novelty processing, as reflected by the affective modulation of the LPP, and that this motivational engagement is only slightly affected by picture repetition. Critically, repetition effects on the LPP affective modulation did not vary as a function of the task, indicating that the evaluative process, and therefore, the engagement of motivational systems, is mandatory. On the other hand, the overall LPP amplitude seems to reflect attention allocation, as it is not only enhanced for novel and target stimuli, but also shows a slower habituation pattern during the categorization task compared to the free-viewing condition, similar to the P3 during active and passive oddball paradigms. The dissociation between the overall LPP amplitude and its modulation as a function of stimulus emotion was also evident in other previous studies. For example, directing attention toward a specific picture category, and thus making it a target, increased the overall amplitude of the LPP, leaving its affective modulation nearly unaffected by target status (Ferrari et al., 2008; Weinberg et al., 2012). Similarly, a reduction in picture size as well as picture exposure duration diminished the overall magnitude of the LPP, but did not dampen the difference in the LPP amplitude between emotional and neutral contents (Codispoti et al., 2009, 2012; De Cesarei and Codispoti, 2011a). Moreover, a previous study by Weinberg and Hajcak (2011) found that the LPP amplitude correlated with RTs in a competing task, whereas the LPP affective modulation was not associated with behavioral emotional interference (i.e., slower RTs for emotional compared to neutral stimuli).

Taken together, these findings are consistent in showing that the enhanced LPP for emotional compared to neutral pictures reflects automatic engagement of motivational systems, which occurs regardless of stimulus novelty and task context (Codispoti et al., 2016; Ferrari et al., 2017b). On the other hand, most of the studies have investigated affective habituation of centrally presented pictures, which may somehow facilitate their affective evaluation, thus prompting LPP modulation. A different scenario, in which emotional cues serve as distractor stimuli and appear displaced in space compared to the target, may reveal different mechanisms that regulate the affective habituation processes. Future studies should examine the extent to which other top-down mechanisms (e.g., spatial attention) might affect emotional habituation.

## Author Contributions

MC and VF designed the research; VF and SM performed the research; VF, SM, and MC analyzed the data; VF, SM, and MC wrote the paper.

## Conflict of Interest Statement

The authors declare that the research was conducted in the absence of any commercial or financial relationships that could be construed as a potential conflict of interest.

## Footnotes

1 International Affective Picture System (Lang et al., 2008) catalog numbers are: Pleasant: 2530, 2550, 4597, 4599, 4601, 4604, 4607, 4608, 4609, 4611, 4616, 4619, 4622, 4623, 4624, 4625, 4626, 4640, 4641, 4643, 4645, 4647, 4649, 4650, 4651, 4652, 4653, 4656, 4658, 4659, 4660, 4664.1, 4666, 4668, 4669, 4670, 4672, 4676, 4677, 4680, 4681, 4683, 4687, 4689, 4690, 4692, 4693, 4694, 4695, 4697, 4698, 4800, 4810. Neutral: 2102, 2104, 2191, 2305, 2372, 2374, 2377, 2382, 2383, 2384, 2390, 2393, 2394, 2400, 2411, 2435, 2485, 2488, 2489, 2495, 2513, 2570, 2579, 2595, 2600, 2635, 2745.1, 2749, 2850, 4605, 5410, 7493, 9331. Unpleasant: 3000, 3010, 3015, 3016, 3017, 3030, 3051, 3053, 3059, 3060, 3061, 3062, 3063, 3064, 3068, 3069, 3071, 3080, 3100, 3101, 3102, 3103, 3110, 3120, 3130, 3131, 3140, 3150, 3160, 3168, 3170, 3180, 3181, 3185, 3190, 3191, 3195, 3212, 3213, 3225, 3261, 3266, 3301, 3400, 6200, 6210, 6212, 6213, 6220, 6231, 6242, 6243, 6244, 6250.1, 6260, 6300, 6312, 6313, 6314, 6315, 6350, 6360, 6410, 6510, 6520, 6530, 6540, 6550, 6555, 6560, 6563, 6570, 6825, 6834, 7062, 7205, 9253, 9405, 9432.
